# Gout and the risk of Parkinson’s disease in older adults: a study of U.S. Medicare data

**DOI:** 10.1186/s12883-018-1234-x

**Published:** 2019-01-05

**Authors:** Jasvinder A. Singh, John D. Cleveland

**Affiliations:** 1 Medicine Service, VA Medical Center, 700 19th St S, Birmingham, Birmingham, AL 35233 USA; 2 Department of Medicine at School of Medicine, University of Alabama at Birmingham, Faculty Office Tower 805B, 510 20th Street S, Birmingham, AL 35294-0022 USA; 3 Division of Epidemiology at School of Public Health, University of Alabama at Birmingham, 1720 Second Ave. South, Birmingham, AL 35294-0022 USA

**Keywords:** Parkinson’s disease, Gout, Association, Medicare, Older adults

## Abstract

**Background:**

In the presence of limited available data, our objective was to assess the association of gout with the risk of incident Parkinson’s disease (PD) in adults 65 years or older.

**Methods:**

We used the 5% random sample of Medicare claims data from 2006 to 2012 to examine the association of gout with incident PD. The multivariable Cox regression model adjusted for demographics, comorbidity, and common cardiovascular disease and gout medications. We calculated hazard ratios (HR) and 95% confidence interval (CI). Sensitivity analyses adjusted for comorbidity categorically, or individually and for additional cardiovascular comorbidities.

**Results:**

In a cohort study, 1.72 million Medicare beneficiaries were eligible. The mean age was 75 years (standard deviation [SD], 7.6), 58% were female, 86% were White and 37% had Charlson-Romano comorbidity index score of ≥2. We found that 22,636 people developed incident PD, 1129 with gout and 21,507 without gout. The respective crude incidence rates of incident PD were 3.7 vs. 2.2 per 1000 person-years. We found that gout was associated with 1.14-times higher hazard ratio (95% CI, 1.07, 1.21) of PD in the main analysis; findings were confirmed in sensitivity analyses. We noted that the risk differed slightly by age; ages 65–75, 75–85 and > 85 had hazard ratios of incident PD with gout of 1.27 (95% CI, 1.16, 1.39), 1.07 (95% CI, 0.97, 1.16) and 0.97 (95% CI, 0.79, 1.20), respectively, but no gender or race differences were noted.

**Conclusions:**

Gout was associated with a higher risk of incident PD in older adults, with the risk being significant in the age group 65–75 years. Future studies need to assess the mechanisms of this increased risk.

**Electronic supplementary material:**

The online version of this article (10.1186/s12883-018-1234-x) contains supplementary material, which is available to authorized users.

## Background

Parkinson’s disease (PD) is a progressive disorder of the nervous system that affects movement, due to the loss of dopaminergic-producing neurons in substantia nigra [1]. PD is the second most common neurodegenerative disorder after Alzheimer’s disease [2] that affects 1% of people older than 60 years and the incidence increases with age [3]. Epidemiological studies have identified few potential risk factors including herbicide or pesticide exposure [4] and hypertension [5]; and an inverse association with current smoking [6] and statin use [7, 8] was noted. Epidemiological data in PD are limited with significant methodological limitations [2]. Due to the low prevalence of PD, many observations have been made using administrative databases [9].

An anti-oxidant effect of urate has been demonstrated in in vivo and in vitro studies [10, 11]. The risk of PD was reported to be decreased in people with higher vs. lower serum urate (sUA) in the general population in two studies [12, 13], but in both studies the association was not significant in the main analysis. Thus, at best both studies gave at least one negative result for each positive association of higher sUA with PD, raising some doubts about such as association.

Whether such hypothesized association of sUA with PD also exist in people with gout, who have much higher levels of sUA that have been shown to be pathogenic, is unknown. Inflammatory arthritis conditions, such as gout, common in the elderly, are associated with increased oxidative stress and chronic inflammation [14], that can potentially increase the risk of PD [15–17]. Acute and chronic inflammatory state in gout might potentially negate the anti-oxidant effects of urate, if one exists physiologically. A recent UK study showed that gout was associated with a 11% increased risk of subsequent PD, largely attributable to an increased risk observed in the early years after hospitalisation for gout, but not after 5 years [18]. In contrast, gout was associated with a 30% reduction in relative risk of PD in a multivariable-adjusted analyses of Canadians 65 years or older [19]. Based on these two cohort studies and three case-control studies, a recent systematic review and meta-analysis, reported a non-significant association of gout with the occurrence of a new diagnosis of PD with a pooled risk ratio of 0.93 (95% CI, 0.79 to 1.09) [20]. However, statistical heterogeneity was high at 87–96%, indicating that studies differed from each other [20]. Thus, it is not clear that gout is associated with PD. If gout is associated with PD, there is no clarity with regards to the direction of this association or the magnitude of the increase/decrease in the risk. Not all studies accounted for all risk factors of PD (hypertension, hyperlipidemia, statin use). Therefore, studies are needed to address this important question. We examined this question in a cohort of older adults, who are at risk of PD [3]. Specifically, we examined whether: (1) gout was associated with an increased risk of PD in older adults; and (2) whether the increased risk differed by key patient demographic characteristics.

## Methods

### Data source and study population

We obtained the 5% Medicare data from 2006 to 2012 from Centers for Medicaid and Medicare (CMS) Chronic Condition Data Warehouse. The 5% random sample Medicare file is a standard Medicare analytic file available from CMS data warehouse that is commonly used to address epidemiological and outcomes questions [21–23]. The advantage is its affordability with limited funds compared to 100% Medicare data. The Institution Review Board at the University of Alabama at Birmingham approved the study (IRB X120207004). We obtained the following data for each Medicare beneficiary: (1) Beneficiary summary file: demographics (birthdate, death date, sex, and race) and monthly entitlement indicators (A/B/C/D); (2) Medicare part A and B files: all inpatient and outpatient claim files including diagnosis codes and claim dates; and (3) Medicare part D file: medication prescription claims (including dose, supply, and drug name) for all filled medications.

We defined the following inclusion criteria for our retrospective cohort study, which is reported according to the Strengthening of Reporting in Observational studies in Epidemiology (STROBE) statement [24]. Patients were included in our study if they were Medicare beneficiaries enrolled in Medicare fee-for-service (Parts A, B) and resided in the U.S. during the study period 2006–2012. CMS generally does not receive claims data for Medicare beneficiaries enrolled in Medicare Advantage Plan (part C) plans. CMS recommends excluding people with Part C claims for Medicare studies [25]. Therefore, we excluded beneficiaries enrolled in Medicare Part C.

### Outcome of interest

Incident PD was identified by the new occurrence of at least two claims for PD at least 4 weeks apart, identified by the presence of an International Classification of Diseases, ninth revision, common modification (ICD-9-CM) diagnostic code, 332.xx, with no previous diagnostic code for PD in the baseline 365-day period (1/1/2005 to 12/31/2005). This algorithm has high accuracy in people 65 years or older, with sensitivity, specificity, positive and negative predictive values of 74.9, 99.8, 80.8 and 99.7%, respectively [26].

### Predictors and covariates

Our main predictor of interest was a diagnosis of gout, classified as two claims of ICD-9-CM diagnostic codes, 274.xx. Patients met the definition on the date of the occurrence of the second code for gout. This approach to identify gout based on ICD-9 codes has been shown to have sensitivity of 90% and specificity of 100% [27].

We included several important covariates and potential confounders, including known risk factors for PD (age, sex, hypertension, hyperlipidemia, statin use) in our analyses. Demographic variables included age, gender, race/ethnicity. Comorbidity was assessed using the Charlson-Romano index, a validated comorbidity index developed for the claims data [28]. It is a weighted comorbidity index consisting of comorbidities such as myocardial infarction, heart failure, liver disease, pulmonary disease, renal disease, etc. We included medications commonly used for the treatment of cardiovascular diseases, namely, statins, beta-blockers, diuretics, and angiotensin converting enzyme (ACE)-inhibitors, to adjust for unmeasured confounding related to cardiovascular disease that could potentially contribute to the risk of PD, and recently described protective effect of statins on the risk of PD [7]. In addition, we also adjusted for commonly used urate-lowering medications for gout, allopurinol and febuxostat, to adjust for potential independent effect of these drugs.

### Statistical analyses

We compared characteristics of patients who developed incident PD during the follow-up to those who remained disease-free during the study period, using a t-test or a chi-square test. We used a multivariable-adjusted Cox proportional hazard regression to assess the independent association of gout with incident PD, adjusted for age, gender, race, Charlson-Romano comorbidity score, cardiovascular medications (statins, beta-blockers, diuretics, ACE-inhibitors) and gout medications (allopurinol, febuxostat; model 1). We calculated hazard ratios (HR) with 95% confidence intervals. We performed sensitivity analyses to test the robustness of our results by replacing the continuous Charlson-Romano score with: (1) categorical variable (score of 0, 1 or ≥ 2; model 2); and (2) individual Charlson-Romano score comorbidities, hypertension, hyperlipidemia and coronary artery disease (model 3). In subgroup analyses, we examined the association of gout with PD by age, sex and race/ethnicity.

## Results

### Study population and crude incidence rate of PD

The overall sample consisted of 1.72 million people, 1.63 million without gout and 94,133 with gout. Mean age was 75.3 years (standard deviation [SD], 7.6), mean Charlson-Romano comorbidity index score was 1.6 (SD, 2.4), 58% were female, 86% were White and 37% had a Charlson-Romano comorbidity index score of ≥2 (Table 1).

**Table 1:** Demographic and clinical characteristics of the study cohort with or without episodes of Incident Parkinson’s Disease

	Entire cohort	Parkinson’s Disease during the follow-up	
		No	Yes
Total people, *N*	1,725,833^a^	1,703,197	22,636
Age, mean (SD)	75.3 (7.6)	**75.3 (7.6)**	**76.5 (6.7)**
Gender, *N* (%)			
Male	730,850 (42.3%)	**719,315 (42.2%)**	**11,535 (51.0%)**
Female	994,983 (57.7%)	**983,882 (57.8%)**	**11,101 (49.0%)**
Race/Ethnicity, *N* (%)			
White	1,486,250 (86.1%)	**1,466,103 (86.1%)**	**20,147 (89.0%)**
Black	142,086 (8.2%)	**140,801 (8.3%)**	**1285 (5.7%)**
Other/unknown	97,497 (5.6%)	**96,293 (5.7%)**	**1204 (5.3%)**
Charlson			
0	909,974 (52.7%)	**901,096 (52.9%)**	**8878 (39.2%)**
1	173,518 (10.1%)	**170,653 (10.0%)**	**2865 (12.7%)**
≥ 2	642,341 (37.2%)	**631,448 (37.1%)**	**10,893 (48.1%)**
Charlson-Romano comorbidity score, mean (SD)	1.60 (2.39)	**1.59 (2.39)**	**2.10 (2.51)**
Charlson-Romano comorbidities			
Myocardial Infarction	68,537 (4.0%)	**67,469 (4.0%)**	**1068 (4.7%)**
Heart Failure	202,196 (11.7%)	**198,932 (11.7%)**	**3264 (14.4%)**
Peripheral vascular disease	167,945 (9.7%)	**164,946 (9.7%)**	**2999 (13.2%)**
Cerebrovascular disease	167,247 (9.7%)	**163,696 (9.6%)**	**3551 (15.7%)**
Dementia	75,936 (4.4%)	**73,689 (4.3%)**	**2247 (9.9%)**
Chronic pulmonary disease	269,751 (15.6%)	**265,719 (15.6%)**	**4032 (17.8%)**
Connective tissue disease	47,999 (2.8%)	**47,267 (2.8%)**	**732 (3.2%)**
Peptic ulcer disease	32,624 (1.9%)	**32,018 (1.9%)**	**606 (2.7%)**
Mild liver disease	8506 (0.49%)	8403 (0.49%)	103 (0.46%)
Diabetes	319,089 (18.5%)	**313,715 (18.4%)**	**5374 (23.7%)**
Diabetes with end organ damage	94,120 (5.5%)	**92,363 (5.4%)**	**1757 (7.8%)**
Hemiplegia	14,118 (0.82%)	**13,833 (0.81%)**	**285 (1.3%)**
Renal failure/disease	59,340 (3.4%)	**58,427 (3.4%)**	**913 (4.0%)**
Any tumor, leukemia or lymphoma	173,685 (10.1%)	**170,940 (10.0%)**	**2745 (12.1%)**
Moderate/severe liver disease	1992 (0.12%)	1959 (0.12%)	33 (0.15%)
Metastatic cancer	17,950 (1.0%)	**17,796 (1.0%)**	**154 (0.68%)**
AIDS	547 (0.03%)	541 (0.03%)	6 (0.03%)
Hypertension	833,128 (48.3%)	**819,889 (48.1%)**	**13,239 (58.5%)**
Hyperlipidemia	601,188 (34.8%)	**591,507 (34.7%)**	**9681 (42.8%)**
Coronary artery disease	302,982 (17.6%)	**297,391 (17.5%)**	**5591 (24.7%)**

^a^ met eligibility criteria and did not have PD in the baseline 365-day period; SD, standard deviation; Bold represents statistically significant differences with *p* <0.001

In our study cohort, 22,636 developed incident PD during the study follow-up, 21,507 in people without gout and 1129 in people with gout. The mean (SD) time from diagnosis of gout to incident PD was 2.38 (SD, 1.76) years (median, 2.1 years; Interquartile range, 0.8, 3.7). In unadjusted analyses, people who developed incident PD were older, and more likely to be male or white. Compared to persons without PD, individual Charlson-Romano comorbidities were more common in people with PD with few exceptions; AIDS, metastatic cancer, and mild liver disease (Table 1).

Crude incidence rate of PD was higher in people with gout compared to those without gout, 3.7 vs. 2.2 per 1000 person-years, respectively (Table 2).

**Table 2:** Crude Incidence rate of Parkinson’s Disease

	Person-months of follow up	#Cases of Parkinson’s Disease	Parkinson’s Disease Incidence Rate per 100,000 person-months	Parkinson’s Disease Incidence Rate per 1000 person-years
Gout	3,649,618	1129	30.9	3.71
No Gout	119,341,989	21,507	18.0	2.16

Incident Parkinson’s Disease was defined as a new diagnosis of PD with an absence of PD diagnosis in the baseline 365-day period, 1/1/2005 to 12/31/2005

### Multivariable-adjusted association of gout with Parkinson’s Disease

Gout was associated with a higher risk of PD in the main analysis, 1.14 (95% CI, 1.07, 1.21) (Table 3). Older age, male gender, White race, and higher Charlson-Romano comorbidity index score were associated with a higher risk of PD (Table 3).

**Table 3:** Association of gout and other risk factors with Incident Parkinson’s Disease, in multivariable-adjusted models, adjusted for demographic factors, hypertension, hyperlipidemia, coronary artery disease, the use of common cardiovascular disease and urate-lowering medications, and Charlson-Romano score as a score (model 1), categorical variable (model 2), or individual comorbidities (model 3)

	Multivariable-adjusted (Model 1)		Multivariable-adjusted (Model 2)		Multivariable-adjusted (Model 3)	
	HR (95% CI)	*P*-value	HR (95% CI)	*P*-value	HR (95% CI)	*P*-value
Age (in years)						
65 - < 75	Ref		Ref		Ref	
75 - < 85	**1.87 (1.82, 1.93)**	**< 0.0001**	**1.86 (1.81, 1.92)**	**< 0.0001**	**1.76 (1.71, 1.81)**	**< 0.0001**
≥ 85	**2.01 (1.92, 2.09)**	**< 0.0001**	**2.02 (1.93, 2.10)**	**< 0.0001**	**1.73 (1.66, 1.81)**	**< 0.0001**
Gender						
Male	Ref		Ref		Ref	
Female	**0.67 (0.66, 0.69)**	**< 0.0001**	**0.67 (0.65, 0.69)**	**< 0.0001**	**0.64 (0.63, 0.66)**	**< 0.0001**
Race						
White	Ref		Ref		Ref	
Black	**0.68 (0.65, 0.72)**	**< 0.0001**	**0.70 (0.66, 0.74)**	**< 0.0001**	**0.67 (0.63, 0.71)**	**< 0.0001**
Other	**0.88 (0.83, 0.93)**	**< 0.0001**	**0.90 (0.85, 0.96)**	**0.0004**	**0.87 (0.82, 0.92)**	**< 0.0001**
Charlson-Romano score, per unit change	**1.15 (1.14, 1.15)**	**< 0.0001**	**N/A**		**N/A**	
Gout	**1.14 (1.07, 1.21)**	**< 0.0001**	**1.16 (1.09, 1.23)**	**< 0.0001**	**1.13 (1.07, 1.21)**	**< 0.0001**
Charlson-Romano index score						
0	**N/A**		Ref		**N/A**	
1			**1.69 (1.62, 1.76)**	**< 0.0001**		
≥ 2			**2.03 (1.98, 2.09)**	**< 0.0001**		
Charlson-Romano comorbidities						
Myocardial Infarction	**N/A**		**N/A**		**0.85 (0.80, 0.91)**	**< 0.0001**
Heart Failure	**N/A**		**N/A**		**1.17 (1.12, 1.22)**	**< 0.0001**
Peripheral vascular disease	**N/A**		**N/A**		**1.13 (1.08, 1.18)**	**< 0.0001**
Cerebrovascular disease	**N/A**		**N/A**		**1.40 (1.34, 1.45)**	**< 0.0001**
Dementia	**N/A**		**N/A**		**3.47 (3.31, 3.64)**	**< 0.0001**
Chronic pulmonary disease	**N/A**		**N/A**		**1.10 (1.06, 1.14)**	**< 0.0001**
Connective tissue disease	**N/A**		**N/A**		**1.13 (1.05, 1.22)**	**0.001**
Peptic ulcer disease	**N/A**		**N/A**		**1.21 (1.11, 1.31)**	**< 0.0001**
Mild liver disease	**N/A**		**N/A**		0.91 (0.74, 1.12)	0.38
Diabetes	**N/A**		**N/A**		**1.18 (1.14, 1.22)**	**< 0.0001**
Diabetes with end organ damage	**N/A**		**N/A**		**1.21 (1.14, 1.28)**	**< 0.0001**
Hemiplegia	**N/A**		**N/A**		**1.20 (1.07, 1.36)**	**0.003**
Renal failure/disease	**N/A**		**N/A**		1.07 (1.00, 1.14)	0.06
Any tumor, leukemia or lymphoma	**N/A**		**N/A**		**1.12 (1.08, 1.17)**	**< 0.0001**
Moderate/severe liver disease	**N/A**		**N/A**		**1.74 (1.21, 2.50)**	**0.003**
Metastatic cancer	**N/A**		**N/A**		0.97 (0.83, 1.14)	0.71
AIDS	**N/A**		**N/A**		0.99 (0.44, 2.20)	0.98
Hypertension	**N/A**		**N/A**		**1.24 (1.20, 1.28)**	**< 0.0001**
Hyperlipidemia	**N/A**		**N/A**		1.03 (0.99, 1.06)	0.12
Coronary artery disease	**N/A**		**N/A**		**1.18 (1.14, 1.23)**	**< 0.0001**

*HR* Hazard ratio, *CI* confidence interval, *Ref* referent category, *N/A* not applicable; Bold represents statistically significant hazard ratios

Model 1 included Charlson-Romano score as a continuous variable, in addition to demographics, hypertension, hyperlipidemia, coronary artery disease, medications for cardiovascular diseases (statins, beta-blockers, diuretics, ACE-inhibitors) and for urate-lowering therapies for gout (allopurinol, febuxostat);

Model 2 included categorized Charlson-Romano score, in addition to demographics, hypertension, hyperlipidemia, coronary artery disease, medications for cardiovascular diseases and for urate-lowering therapies for gout; and

Model 3 included each of the 17 Charlson-Romano comorbidities in addition to demographics, hypertension, hyperlipidemia, coronary artery disease, medications for cardiovascular diseases and for urate-lowering therapies for gout

Sensitivity analyses that adjusted for a categorical Charlson index or individual Charlson index comorbidities confirmed the findings from the main study, and the respective hazard ratios of gout with incident PD were 1.16 (95% CI, 1.09, 1.23) and 1.13 (95% CI, 1.07, 1.21) (Table 3).

Subgroup analyses by age, sex and race revealed significant differences only by age, with ages 65 - < 75, 75 – < 85 and ≥ 85 years with hazard ratios of incident PD with gout of 1.27 (95% CI, 1.16, 1.39), 1.07 (95% CI, 0.97, 1.16) and 0.97 (95% CI, 0.79, 1.20), respectively (Additional file 1).

## Discussion

In a study of older American adults, we found that gout was independently significantly associated with slightly increased risk of incident (a new diagnosis of) PD. The increase in hazards was independent of age, gender, race, and comorbidities, potential risk factors for PD. In subgroup analyses, we found that the increased risk of PD with gout varied significantly by age. Several findings merit further discussion.

In a UK study, gout was associated with a modest elevation of the overall risk of subsequent PD with a relative risk (RR) of 1.11 (95% confidence interval, 1.05–1.17), attributable to an increased risk observed in the early years after hospitalisation for gout [18]. In contrast, De Vera et al. studied Canadians 65 years or older and reported a protective effect of gout, with a relative risk of 0.70 of PD (95% CI 0.59–0.83), after adjusting for age, sex, comorbid medical conditions, and use of diuretics and NSAIDs [19]. Both studies used administrative databases and defined gout and PD similar to each other and to our study. However, the two studies differed in patient population (all comers vs. ≥65 years), setting (inpatients with gout vs. all with gout), country (UK vs. Canada) and the confounders adjusted in the analyses. A recent systematic review and meta-analysis that included these two cohort studies and three additional case-control studies, found no difference in the pooled risk ratio of PD in patients with gout [20]. The statistical heterogeneity in the meta-analysis was high (87–96%), indicating differences in studies (demographics, inclusion criteria etc.) from each other [20]. Additionally, the risk of PD was decreased in people with higher vs. lower sUA in the general population in two studies [12, 13], but in both studies the association was not significant in the main analysis. There was a difference of only 0.4 mg/dl between the highest vs. the lowest sUA quartiles (5.7 vs. 6.1 mg/dl) [12], and the mean sUA in both quartiles were in the normal range. In the other study, there were no significant differences in PD risk by sUA quartiles, or by sUA level as a continuous variable [13].Therefore, the current evidence regarding gout and the risk of PD is contradictory, and it was unclear as to which study finding represented a true phenomenon. Hence, robust evidence from generalizable populations is needed.

We analyzed the U.S. Medicare data that is representative of Americans 65 years or older, adjusting for statin use and cardiovascular disease, potential confounders of PD risk (and previously not accounted for), and the use of urate-lowering therapy (allopurinol, febuxostat) previously hypothesized (and previously noted) to have an opposite effect to that of gout. A limitation of the previous studies was the lack of adjustment for urate-lowering therapy. De Vera produced separate estimates for those using vs. not using anti-gout treatment, but lumped urate-lowering drugs with colchicine, and these two have different mechanisms of action [19]. None of the previous studies separated the disease effect (gout) from that of the treatment (urate-lowering therapy) and therefore were potentially confounded.

The recent systematic review and meta-analysis that included three case-control studies and two cohort studies showed no increase in subsequent PD in patients with gout, the pooled risk ratio was 0.93 (95% CI, 0.79 to 1.09) [20]. Two major limitations noted by authors and us were the high study heterogeneity in pooled estimate from the meta-analysis and the inclusion of very few studies. To our knowledge, our study among the first few studies to report a robust positive association of gout with PD in multivariable-adjusted analyses that included known PD risk factors.

One of the pathogenic mechanisms of PD is oxidative stress, which is linked to other components of degenerative process, such as mitochondrial dysfunction, inflammation, nitric oxide toxicity and excitotoxic mechanisms [15]. Oxidative damage to lipids, proteins and DNA is evident in PD, [16] and protein degradation associated with oxidative damage is implicated in dopaminergic cell death in PD [17]. Patients with PD have increased levels of oxidized lipids, proteins and DNA and decreased levels of reduced glutathione have been found in substantia nigra, indicative of oxidative stress. Oxidative stress and local inflammation (elevated inflammatory mediator levels in brain) [29, 30] are linked to neurodegenerative processes in PD that are responsible for the disease and its manifestations. Inflammation (both local and systemic) and increased oxidative stress are hallmarks of gout, a chronic, crystal-induced inflammatory arthritis in adults [14, 31–33]; urate crystals engage caspase-1-activating NLRP3 inflammasome, resulting in the production of interleukin(IL)-1beta and IL-18, pro-inflammatory cytokines [34]. Therefore, we hypothesized that gout would be associated with a higher risk of PD in older Americans. We believe that previous studies that found either no association or a potential beneficial effect of gout on Parkinson’s disease were confounded by the lack of adjustment for risk factors for PD (statin use, cardiovascular comorbidity), and urate-lowering therapy, which is frequently used by patients with gout and has a protective effect regarding PD. We can not rule out the hypothesis that increased risk of PD imparted by gout might differ by the age of onset of PD, since we only studied adults 65 years or older. Future studies need to further assess these issues and underlying mechanisms.

An interesting finding in our study the variation of increased risk of PD with gout by age. We found that the hazards ratios of gout with incident PD revealed a risk-gradient in the age groups, 65 - < 75, 75 – < 85 and ≥ 85 years at 1.27, 1.07 and 0.97, respectively. This indicates that most of the increased risk in the older adults comes from the risk in age group 65- < 75 years, for which it was also statistically significant.

Another interesting finding was that we did not notice any differential risk of PD with gout by gender or race/ethnicity. While both female gender and Black race were protective for the risk of PD (Table 3) as reported previously [35], the hazards ratios of PD with gout did not differ statistically significantly by gender or race/ethnicity (*p* > 0.05 for both interaction terms; Additional file 1). Reported PD incidence rates in our general population without gout are consistent with those reported by National Institutes of Health [36], given that there were 46–54 million Americans in the Medicare program during our study period [37].

Our study has several limitations that must be considered when interpreting findings. Misclassification bias is possible, since we used database diagnosis of PD and gout, and both under-diagnosis and over-diagnosis are possible. Atypical Parkinsonism (multiple system atrophy [MSA], progressive supranuclear palsy [PSP], Lewy body dementia [LBD], corticobasal degeneration [CBD] etc.) may have been misdiagnosed as PD. However, the similarity of incidence rates of PD in our study to those reported in previous studies for this age group [38], including a National Institutes of Health study [36], increased our confidence in findings and indicated that misclassification bias was likely minimal [2]. We used validated algorithms for identifying gout and PD, which have been shown to have high accuracy [26, 27]. We considered examining all incident PD versus only PD subgroups, such as those with vs. without dementia, but examining vascular PD separately was a not a focus of this study. To our knowledge, there are no validation data for identifying this disease subset in a database study such as ours and are no specific ICD-9 codes for this subtype of PD. Future studies can examine these important subgroups as appropriate for study hypotheses. The observational nature of the study puts our finding at the risk of confounding bias, despite our attempt to include several covariates and potential confounders, including medications and disease conditions. We conducted sensitivity analyses by varying the way we included the 17 Charlson comorbidities, which confirmed the findings from the main model with minimal attenuation of hazard ratios. We were unable to assess the role serum urate or inflammatory markers play in this increased risk due to the non-availability of these data in Medicare data. Future studies can address this by using datasets that provide this information. Our study has several strengths, including the use of a representative sample of older American adults, a large sample with enough incident PD cases and the adjustment for several covariates/confounders, including most risk factors for PD.

## Conclusions

In conclusion, we found that gout was associated with a modest increase in the risk of PD for adults 65 years or older as a group, independent of other factors. The increase in PD risk with gout differed by patient age in further analyses by age categories, statistically significant only for the 65- < 75-year age group. Mechanisms of this increased risk of PD in patients with gout needs to be investigated further, and what role inflammation, oxidative stress and serum urate play in this need to be assessed.

## Additional file

Additional file 1: Association of gout with Parkinson’s Disease, in pre-defined subgroup analyses, varying by age, gender and race. This additional file shows the hazard ratios of the association of gout with Parkinson’s Disease, by age, gender and race subgroups. Additional file 1 was presented at the 2018 ACR/ARHP Annual Meeting as part of the meeting abstract and poster. (DOCX 19 kb)

## Abbreviations

ACE inhibitor: Angiotensin converting enzyme inhibitor
CAD: Coronary artery disease
CMS: Centers for Medicare and Medicaid Services
EULAR: European League Against Rheumatism
ICD-9-CM: International Classification of Diseases, ninth revision, common modification
PD: Parkinson’s disease
ULT: urate-lowering therapy
XDH: Xanthine dehydrogenase
XO: Xanthine oxidase
XOR: Xanthine oxido-reductase system
