# Supplementary material for: Gout and the risk of Parkinson’s disease in older adults: a study of U.S. Medicare data
Source: BMC Neurol. 2019 Jan 5;19:4. doi: 10.1186/s12883-018-1234-x (PMC6321725; doi:10.1186/s12883-018-1234-x)
Supplement: Supplementary file 1 — Association of gout with Parkinson’s Disease, in pre-defined subgroup analyses, varying by age, gender and race. This additional file shows the hazard ratios of the association of gout with Parkinson’s Disease, by age, gender and race subgroups. Additional file 1 was presented at the 2018 ACR/ARHP Annual Meeting as part of the meeting abstract and poster. (DOCX 19 kb) [file 12883_2018_1234_MOESM1_ESM.docx]

**Additional Files**

**Additional File 1.** Association of gout with Parkinson’s Disease, in pre-defined subgroup analyses, varying by age, gender and race

|  | **Multivariable-adjusted (Model 1)** | | **Multivariable-adjusted**  **(Model 1)** | | **Multivariable-adjusted**  **(Model 1)** | |
| --- | --- | --- | --- | --- | --- | --- |
|  | HR (95% CI) | P-value | HR (95% CI) | P-value | HR (95% CI) | P-value |
|  | **65-<75 years** | | **75- <85 years** | | **≥85 years** | |
| Gout | **1.27 (1.16, 1.39)** | **<0.0001** | 1.07 (0.97, 1.16) | 0.17 | 0.97 (0.79, 1.20) | 0.77 |
|  |  |  |  |  |  |  |
|  | **Female** | | **Male** | |  |  |
| Gout | **1.17 (1.06, 1.30)** | **0.003** | **1.11 (1.03, 1.20)** | **0.006** |  |  |
|  |  |  |  |  |  |  |
|  | **Black** | | **White** | | **Other race** | |
| Gout | 1.09 (0.87, 1.36) | 0.46 | **1.13 (1.06, 1.21)** | **0.0003** | **1.36 (1.05, 1.77)** | **0.02** |
|  |  |  |  |  |  |  |
| Interaction terms: Gout*age p-value **<0.0001**; Gout*gender p-value = 0.52; Gout*race p-value = 0.65  HR, Hazard ratio; CI, confidence interval;  **Bold estimates represent those with statistical significance, i.e., p-value <0.05** | | | | | | |
